# Potassium intake assessed by dietary recall and urinary excretion in young South African adults: findings from the African-PREDICT study

**DOI:** 10.1186/s12889-026-28605-5

**Published:** 2026-07-21

**Authors:** Arole Shivambu, Antony K. Chiromba, Tertia van Zyl

**Affiliations:** 1 Centre of Excellence for Nutrition, Faculty of Health Sciences, North-West University, Potchefstroom, South Africa; 2 SAMRC/NWU Extramural Unit for Hypertension and Cardiovascular Disease, Faculty of Health Sciences, North-West University, Potchefstroom, South Africa; 3 Centre of Excellence for Nutrition (CEN), North-West University, 11 Hoffman Street, Potchefstroom, 2531 South Africa

**Keywords:** Potassium intake, Dietary potassium, Urinary potassium, Blood pressure, Young adults

## Abstract

**Background:**

South Africa has strong public health initiatives targeting salt reduction, yet population-level evidence and strategies regarding potassium intake remain limited. This gap is important given the role of potassium in mitigating sodium-related increases in blood pressure and cardiovascular disease risk. This study assessed potassium intake using dietary and urinary measures in young adults from the African-PREDICT study.

**Methods:**

Potassium intake was estimated using three 24-hour dietary recalls and a single 24-hour urine collection per participant. Agreement between methods was evaluated using Bland-Altman analysis.

**Results:**

Median potassium intake was 1911 mg/day from dietary assessment and 1683 mg/day from urinary analysis, both well below the recommended 3510 mg/day level, which is linked to sodium counterbalance and reduced cardiovascular risk. Over 90% of participants failed to meet the recommended intake across methods. Black participants and individuals of low socioeconomic status (SES) had substantially lower potassium intakes than their counterparts. Bland-Altman analysis demonstrated limited individual-level agreement (with a 95% limit-of-agreement span of approximately 5,600 mg/day), with dietary potassium intake marginally higher than urinary excretion (mean difference = 61 mg/day; *p* = 0.19), reflecting expected physiological potassium handling. In adjusted regression analyses, dietary potassium was inversely associated with diastolic blood pressure, while both urinary potassium and the sodium-to-potassium ratio showed no significant associations.

**Conclusion:**

Both methods show that potassium intake is low among young adults in South Africa, particularly among Black and low-SES groups, highlighting the need for population-level strategies to increase potassium consumption, particularly in vulnerable groups. The sodium-to-potassium ratio was associated with blood pressure only in the unadjusted model, whereas dietary potassium was associated with diastolic blood pressure in the adjusted model. Dietary assessment may be appropriate for population-level estimation of potassium intake, but its limited agreement with urinary potassium excretion at the individual level, particularly at higher intakes, may limit its utility for individual assessment.

**Supplementary Information:**

The online version contains supplementary material available at https://doi.org/10.1186/s12889-026-28605-5.

## Introduction

Hypertension remains a major contributor to cardiovascular diseases (CVD) and global premature mortality [1]. In South Africa, CVD accounts for over 17.6% of deaths [2], while hypertension prevalence exceeds 40% [3], placing a substantial economic burden on the South African healthcare system [4]. Excessive salt intake remains one of the key modifiable risk factors for hypertension [5], yet South Africans continue to consume salt at levels (> 5 g/day) linked to elevated blood pressure and hypertension [6, 7]. Young adulthood is a critical period for cardiovascular risk modification, as dietary patterns established during this life stage often track into later adulthood [8].

Adequate potassium intake (> 3510 mg/day) is essential for cardiovascular health, as this level counteracts the blood pressure-raising effects of high sodium intake [9]. Fruits and vegetables are a primary dietary source of potassium [10], yet intake in South Africa remains consistently low [11]. Other good dietary sources of potassium include food groups like legumes, whole grains, and dairy products such as yoghurt [12]. Previous cohort studies report that over 90% of South African adults [13] and primary school children [14] have inadequate potassium intake levels.

Although South Africa has made significant progress in implementing population-level salt reduction strategies [15, 16], efforts specifically aimed at improving dietary potassium remain limited. Increasing potassium intake represents a promising and cost-effective approach for mitigating hypertension and reducing CVD risk in South Africa [13]. The recent interest in potassium-enriched low-sodium salt substitutes in South Africa further highlights the growing focus on potassium-based interventions [17, 18]. However, the potential impact of such strategies cannot be meaningfully evaluated without robust, up-to-date data on potassium intake patterns across South African population subgroups.

To address this evidence gap, this study evaluated potassium intake among young adults in the African-PREDICT cohort using both repeated 24-hour dietary recalls and 24-hour urinary potassium excretion. The study also aimed to compare these two potassium assessment methods to assess their consistency and reliability. Agreement between dietary potassium intake and 24-hour urinary potassium excretion was evaluated to assess the validity of the dietary method relative to urinary excretion, the reference method for population-level estimation of potassium intake. However, a single 24-hour urine collection is subject to within-person variability and may not fully capture usual potassium intake [19–21].

This comparison was conducted to determine whether dietary recalls, being more feasible and cost-effective [22] can serve as a reliable tool for large-scale potassium intake surveillance, particularly in low- to middle-income settings such as South Africa. Potassium intake patterns were described by race, sex, and socioeconomic status (SES).

## Method

### Study design, setting, and participants

**Fig. 1:**
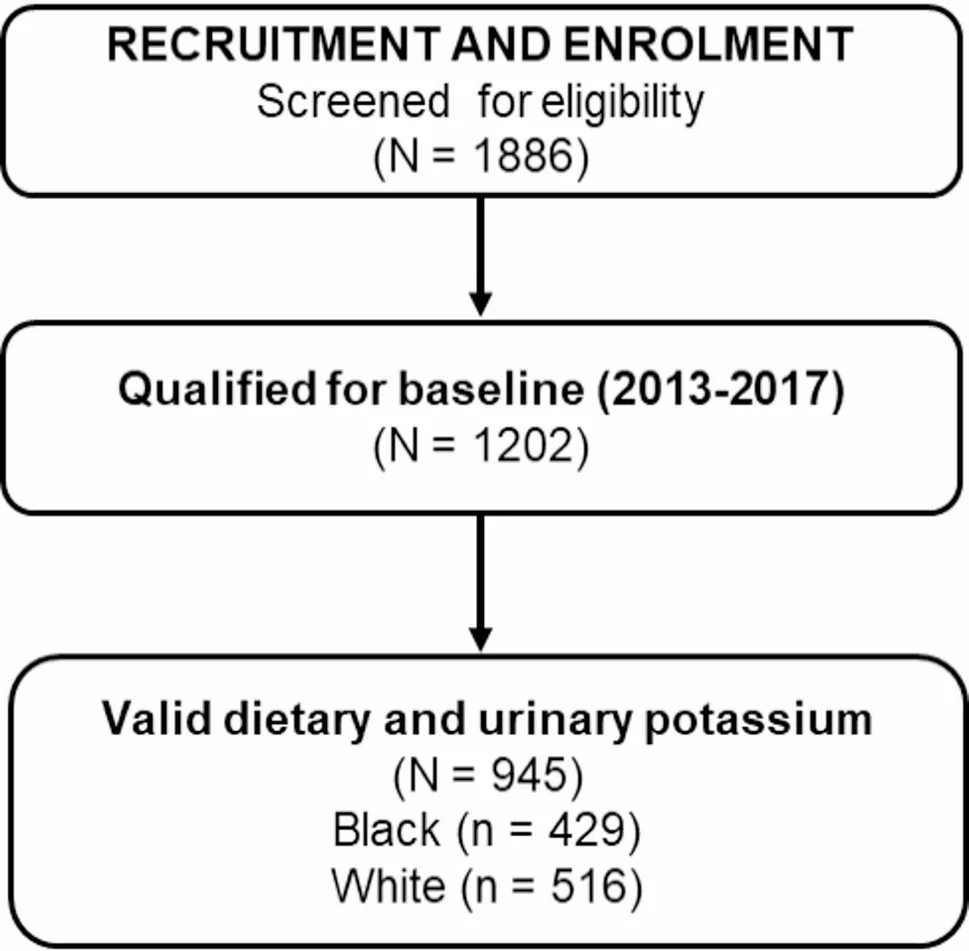
Flow chart showing baseline recruitment and enrolment of the African-PREDICT study

Figure 1 displays the recruitment and enrolment of baseline participants in the African-PREDICT study. Study participants were recruited between October 2012 and December 2017 in and around the Potchefstroom area of the North West Province, during which 1,200 individuals were enrolled. Recruitment was conducted through a combination of active community engagement, workplace-based recruitment, health screening initiatives in public spaces, and public advertising in local newspapers, on noticeboards, and on the radio, to ensure representation across socioeconomic strata. Screening was conducted either on-site or at the research facility. The research team coordinated all recruitment procedures, and participation was voluntary [23].

The African-PREDICT study is an ongoing 10-year longitudinal study that aims to identify early markers and predictors of CVD development in a sample of apparently healthy young adults aged 20–30 years. The study includes men and women from black and white races. The study involved normotensive individuals without chronic diseases, not taking any chronic medications, and not pregnant or breastfeeding. Detailed information on the inclusion and exclusion criteria is described elsewhere [23]. For this study, the selection criteria excluded participants with missing dietary and urinary data, resulting in a sample of 945. We assessed potential exclusion-related bias by comparing the characteristics of participants in the final analytic sample with those excluded because of incomplete dietary and urinary potassium data (Supplementary Table S1).

### Dietary data collection

Dietary intake was assessed using three 24-hour dietary recalls conducted on non-consecutive days, including one weekend day, for each participant. The first recall was administered on-site by a trained nutritionist. At the same time, the remaining two were conducted off-site at participants’ homes or another convenient location using the same standardized procedures. Participants were required to report all foods and beverages consumed during the preceding 24 h. Detailed information collected included the time and place of consumption, food descriptions, preparation methods, ingredients, and brand names. Portion sizes were estimated using standardized tools, such as household measures, plastic food models, and photographic aids, to improve accuracy [23]. All completed recalls were reviewed, coded according to the South African Medical Research Council (SAMRC) Food Quantity Manual and SAMRC Food Composition Tables to convert household measures into grams. The recorded dietary data were subsequently analysed for nutrient content by the SAMRC [24].

### 24-hour urine collection and urinary potassium and sodium analysis

Participants collected urine over a 24-hour period using a one-litre jug and transferred it into a five-litre container, which was stored in a cool, dark place until retrieval. Samples were transported to the laboratory, aliquoted into four 1.5-ml tubes, labelled, and stored at − 20 °C until analysis. Detailed information on 24-hour urine collection is explained elsewhere [23].

Potassium and sodium in the urine were determined using an Ion-Selective Electrode (indirect) with a Beckman Coulter Synchron CX5PRO System. The sodium: potassium ratio was derived by dividing total 24-hour urinary sodium excretion (mmol/day) by total 24-hour urinary potassium excretion (mmol/day), while salt intake in grams per day was derived by dividing total 24-hour urinary sodium excretion (in mmol/day) by 16.964, which represents the molecular weight based conversion factor used to express sodium excretion as sodium chloride [23].

### Blood pressure measurements and anthropometric measurements

Blood pressure was measured using the Dinamap ProCare 100 Vital Signs Monitor (GE Medical Systems, Milwaukee, USA); the measurements were conducted by trained research staff under standardized conditions. Participants were required to refrain from eating, smoking, or exercising for at least 30 min before measurements were taken on the less active arm, supported at heart level, with three readings obtained at five-minute intervals, and the average of these measurements was used. Systolic and diastolic blood pressure values were recorded [23].

Weight was measured to the nearest 0.01 kg using SECA electronic scales (SECA, Birmingham, UK), and height to the nearest 0.1 cm using a SECA stadiometer; body mass index (BMI) was calculated as kg/m². Waist circumference was measured in triplicate with a non-flexible tape measure (Holtain, Crymych, UK) and recorded to the nearest 0.1 cm [23].

### Covariates

General health and demographic information, was collected using a validated questionnaire [25], including information on physical activity level [26]. SES was assessed using the Kuppuswamy Socioeconomic Scale, originally developed and validated in India [25]. For the African-PREDICT study, SES was determined using a point-based scoring system derived from the scale, incorporating the occupation, monthly household income, and education level of the household head. Participants were subsequently classified into three SES categories: low, middle, and high, as described elsewhere [27]. Alcohol intake was measured with a self-reported general health questionnaire that captured regular alcohol consumption, beverage type, and quantity and frequency of intake per week using predefined categorical responses [23].

#### Clinical trial number

Not applicable.

### Statistical analysis

The statistical tests were performed using the IBM SPSS Statistics, Version 30.0 (IBM Corp., Armonk, NY, USA). Normality testing was done visually using histograms. Potassium intake was assessed using descriptive statistics, and the data are reported as the mean and standard deviation or the median and interquartile range (25th-75th percentiles), depending on the normality. The sample size was determined by the number of participants with complete dietary and 24-hour urinary data available in the African-PREDICT study.

The agreement between dietary and urinary assessment methods was tested using a Bland-Altman plot method. Furthermore, proportional bias was evaluated using linear regression of the difference between methods (dietary potassium minus urinary potassium) against the mean of the two measurements, following Bland-Altman methodology, with analyses performed on untransformed data.

The association between potassium intake and blood pressure was evaluated using multiple linear regression, while adjusting for age, sex, ethnicity, BMI, SES, urine sodium excretion, total energy intake, alcohol intake, and fruit and vegetable consumption. Additionally, potassium intake variables, including the Na: K ratio, were log-transformed before analysis to improve normality and model fit.

## Results

Participant characteristics are detailed in Table 1. The average BMI among all participants was 25.2 ± 5.7 kg/m², with a systolic blood pressure of 118.8 ± 11.6 mmHg. The median fruit and vegetable intake was 166 g/day (92–284). The median dietary potassium intake was 1911 mg/day (1349–2560), while the median 24-hour urinary potassium excretion was 1683 mg/day (1142–2533). Median sodium excretion was 3170 mg/day (2187–4457), equating to an average salt intake of 9.3 ± 6.1 g/day. The mean urine sodium-to-potassium ratio was 3.64 ± 2.25.

For racial comparison, Black participants had a lower BMI (24.8 ± 5.9 vs. 25.5 ± 5.4 kg/m²) and a slightly higher mean systolic blood pressure, although the values are largely comparable (119.6 ± 11.5 vs. 118.1 ± 11.7 mmHg). Fruit and vegetable intake was lower among Black participants (148 g/day) compared to their White counterparts (187 g/day). Both dietary potassium intake (1455 vs. 2272 mg/day) and urinary potassium excretion (1315 vs. 1980 mg/day) were lower in Black compared with White participants. Salt intake was similar between groups (9.5 vs. 9.2 g/day), although the urine sodium-to-potassium ratio was higher in Black participants (4.38 vs. 3.01).

**Table 1:** Characteristics of participants by race

Characteristic	All participants (*N* = 945)	Black (*n* = 429)	White (*n* = 516)
Demographic and anthropometric			
Age (years)	24.5 (3.1)	24.4 (3.1)	24.5 (3.1)
Body mass index (kg/m²)	25.2 (5.7)	24.8 (5.9)	25.5 (5.4)
Waist circumference (cm)	80.6 (12.7)	78.4 (11.1)	82.4 (13.7)
Socioeconomic status			
Low, n (%)	352 (37.2)	247 (57.6)	105 (20.3)
Middle, n (%)	279 (29.5)	119 (27.7)	160 (31.0)
High, n (%)	314 (33.2)	63 (14.7)	251 (48.6)
Blood pressure			
Systolic blood pressure (mmHg)	118.8 (11.6)	119.6 (11.5)	118.1 (11.7)
Diastolic blood pressure (mmHg)	78.5 (7.4)	79.6 (7.8)	77.6 (6.8)
Dietary intake			
Fruit and vegetables (g/day)†	166 (92–284)	148 (83–249)	187 (99–312)
Dietary potassium (mg/day)	1911 (1349–2560)	1455 (1122–1958)	2272 (1749–2813)
Total energy intake (kJ/day)	6992 (2894)	6846 (2903)	7118 (2883)
Urinary measures			
24-h urinary potassium (mg/day)†	1683 (1142–2533)	1315 (908–2032)	1980 (1424–2797)
24-h urinary sodium (mg/day)†	3170 (2187–4457)	3187 (2212–4680)	3154 (2173–4332)
Salt intake (g/day)	9.3 (6.1)	9.5 (5.7)	9.2 (6.5)
Urinary sodium-to-potassium ratio	3.64 (2.25)	4.38 (2.11)	3.01 (2.17)
Urine volume (L)†	1.22 (0.88–1.71)	1.17 (0.85–1.67)	1.28 (0.92–1.76)

Table Caption: Values are presented as mean (SD) unless otherwise indicated. † Median (interquartile range). Socioeconomic status was classified according to predefined criteria described in the Methods section. Race was self-identified. The table is descriptive; no statistical comparisons are shown. Salt intake was estimated from urinary sodium excretion

### Daily potassium intake according to 24-hour dietary recalls (dietary potassium) and 24-hour urine (urinary potassium)

Table 2 details potassium intake based on 24-hour dietary recalls (dietary potassium) and 24-hour urine collections (urinary potassium), stratified by race, sex, and SES. Among all participants, the median dietary potassium intake was 1911 mg/day (IQR: 1349–2560), while the median urinary potassium excretion was 1683 mg/day (IQR: 1142–2533). The difference between dietary intake and urinary excretion was statistically significant (Z = − 3.91; *p* < 0.001). Only a small proportion of participants met the daily recommended potassium intakes based on dietary assessment (7%) and urinary potassium (9.5%).

**Table 2:** Comparison of dietary and urinary potassium intake by socio-demographic sub-groups

Group		Dietary potassium (mg/day) Median (IQR)	24-h urinary potassium (mg/day) Median (IQR)	Z-value	*P*-value	Meeting recommended intake, *n* (%) Dietary | Urinary
All participants (*N* = 945)		1911 (1349–2560)	1683 (1142–2533)	-3.91	< 0.001	68 (7.0) | 90 (9.5)
Ethnicity	Black	1455 (1122–1958)	1316 (908–2032)	-1.82	0.069	11 (2.4) | 26 (6.1)
	White	2272 (1749–2813)	1980 (1424–2797)	-3.64	< 0.001	57 (10.9) | 64 (12.4)
Sex	Women	1750 (1293–2369)	1563 (1146–2332)	-2.07	0.038	25 (5.2) | 33 (7.0)
	Men	2071 (1429–2747)	1818 (1138–2706)	-3.38	< 0.001	43 (8.7) | 57 (12.1)
Socioeconomic status groups	Low	1495 (1144–2112)	1370 (947–2199)	-1.03	0.305	15 (4.1) | 31 (8.8)
	Middle	1998 (1400–2571)	1818 (1280–2759)	-0.79	0.431	26 (9.0) | 37 (13.3)
	High	2240 (1710–2794)	1899 (1330–2580)	-5.01	< 0.001	27 (8.5) | 22 (7.0)

Data are presented as median (IQR). Differences between dietary potassium intake and 24-hour urinary potassium excretion within individuals were assessed using the Wilcoxon signed-rank test. Analyses were conducted for the overall sample and stratified by race, sex, and socioeconomic status. Z-values and corresponding p-values are reported. Meeting the recommended intake is presented as a number (percentage) based on dietary and urinary measures, respectively. Recommended potassium intake is defined according to Aburto et al. [9], however, urinary potassium values were interpreted in relation to this dietary reference as a proxy for intake at the population level, and such comparisons should be made with caution, as urinary excretion does not fully reflect dietary intake [28]

Across race, Black participants had lower dietary potassium intake (1455 mg/day [IQR: 1122–1958]) and urinary potassium excretion (1316 mg/day [IQR: 908–2032]) than White participants (2272 mg/day [IQR: 1749–2813] and 1980 mg/day [IQR: 1424–2797], respectively). Differences between dietary and urinary potassium were not statistically significant among Black participants (Z = − 1.82; *p* = 0.069) but were significant among White participants (Z = − 3.64; *p* < 0.001). Only 2.4% of Black participants met the recommended intake based on dietary assessment and 6.1% based on urine, compared with 10.9% and 12.4% of White participants, respectively.

By sex, men exhibited higher levels of both dietary potassium intake (2071 mg/day [IQR: 1429–2747]) and urinary potassium excretion (1818 mg/day [IQR: 1138–2706]) compared with women, who had lower intake and excretion values (1750 mg/day [IQR: 1293–2369] and 1563 mg/day [IQR: 1146–2332], respectively). Differences between dietary intake and urinary excretion were statistically significant in both men (Z = − 3.38; *p* < 0.001) and women (Z = − 2.07; *p* = 0.038). The proportion meeting potassium intake recommendations was higher in men (8.7% diet; 12.1% urine) than in women (5.2% and 7.0%, respectively).

Dietary potassium intake and urinary potassium excretion increased across SES groups. Participants in the Low SES had the lowest dietary (1495 mg/day [IQR: 1144–2112]) and urinary potassium (1370 mg/day [IQR: 947–2199]), while high SES individuals had the highest (2240 mg/day [IQR: 1710–2794] and 1899 mg/day [IQR: 1330–2580], respectively). Differences between dietary and urinary potassium were significant only in the high SES group (Z = − 5.01; *p* < 0.001).

### Comparison of potassium intake estimates using 24-hour dietary recalls and 24-hour urine

**Fig. 2:**
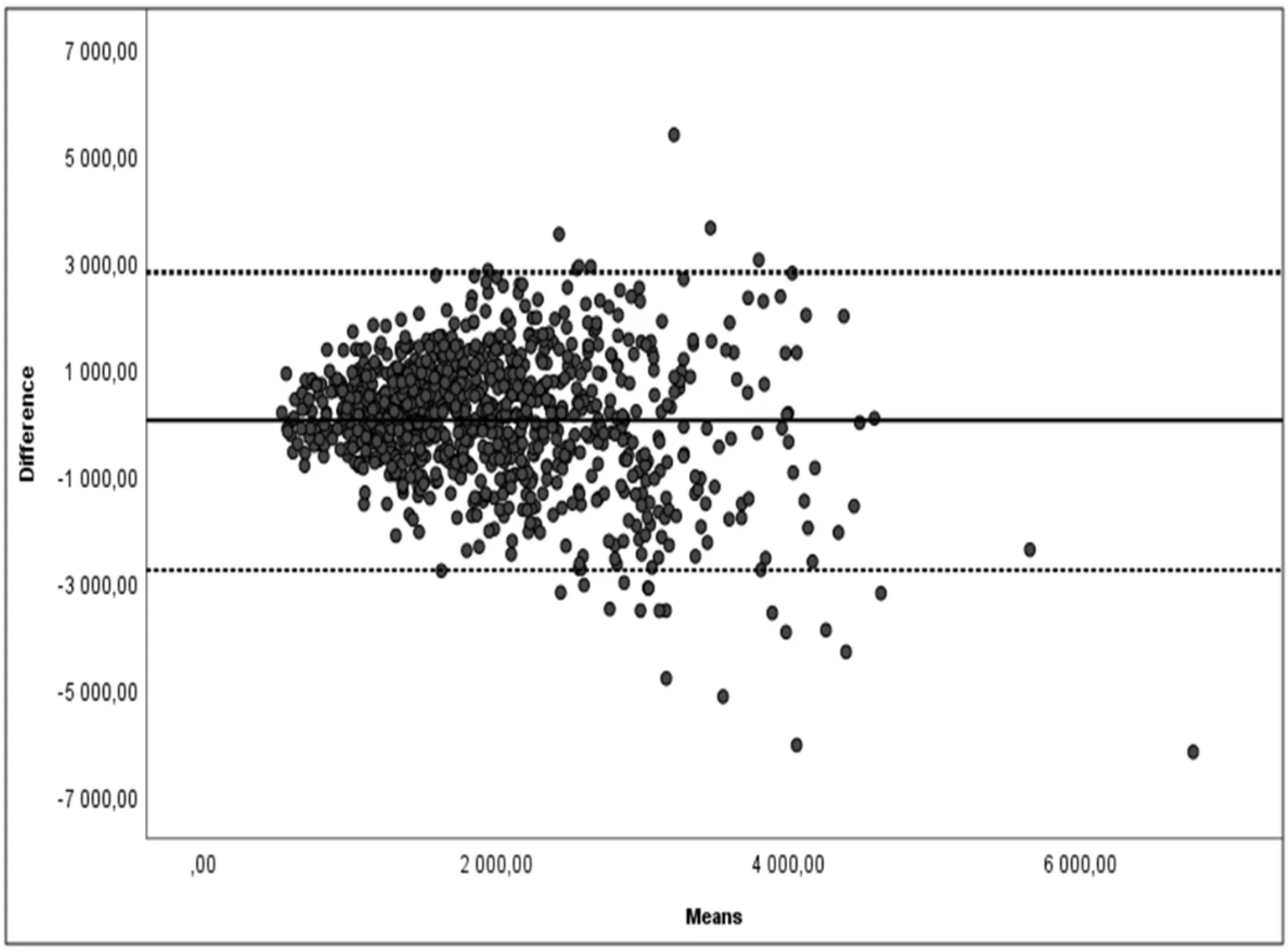
Bland-Altman plot showing the disagreement in potassium intake between dietary assessment and urine analysis methods. The y-axis represents the difference between the two methods (dietary assessment potassium minus urinary potassium). The x-axis represents the mean between the two methods. The middle horizontal line represents the mean difference in potassium values (61.3 mg/day; *p* = 0.19). The upper and lower horizontal lines represent the lower (− 2745.90) and upper (2868.48) limits of agreement

Figure 2 shows the Bland-Altman plot displaying the disagreement between potassium intake levels from dietary assessment (method 1) and urinary assessment (method 2). A significant proportional bias was observed (β = −0.576; *p* < 0.001). The negative slope indicates that as mean potassium intake increases, the difference between the two methods becomes more negative, demonstrating increasing underestimation by method 1 at higher potassium intake levels. These findings suggest that dietary recalls may be suitable for broad population surveillance but are less reliable for estimating absolute values at higher potassium intakes.

### Association of dietary and urinary potassium with brachial blood pressure

In unadjusted linear regression analysis (Table 3), dietary potassium showed no meaningful association with systolic or diastolic blood pressure, with effect estimates close to zero and confidence intervals crossing the null. In contrast, 24-hour urinary potassium demonstrated a positive association with systolic blood pressure (B = 1.227, 95% CI: 0.006 to 2.447, *p* = 0.049), but no association with diastolic blood pressure (B = − 0.115, 95% CI: −0.892 to 0.661, *p* = 0.771). The sodium-to-potassium ratio was positively associated with both systolic (B = 2.372, 95% CI: 0.979 to 3.766, *p* < 0.001) and diastolic blood pressure (B = 1.353, 95% CI: 0.467 to 2.239, *p* = 0.003).

**Table 3:** Association test between potassium intake and brachial blood pressure

Variable	Outcome	Unadjusted B (95% CI)	*p*-value	Adjusted B (95% CI)	*p*-value
Dietary potassium	Systolic	1.344 (− 0.193; 2.881)	0.086	−0.494 (− 2.133; 1.144)	0.554
	Diastolic	−0.813 (− 1.789; 0.164)	0.103	−1.339 (− 2.531; −0.147)	0.028
Urinary potassium	Systolic	1.227 (0.006; 2.447)	0.049	0.476 (− 0.747; 1.699)	0.445
	Diastolic	−0.115 (− 0.892; 0.661)	0.771	−0.164 (− 1.058; 0.729)	0.718
Na: K ratio	Systolic	2.372 (0.979; 3.766)	< 0.001	1.187 (− 0.305; 2.679)	0.119
	Diastolic	1.353 (0.467; 2.239)	0.003	0.789 (− 0.301; 1.879)	0.156

Associations were assessed using linear regression models with blood pressure as a dependent variable. Variables for dietary potassium, urinary potassium, and Na: K ratio were log-transformed before analysis. Values are presented as regression coefficients (B) with 95% confidence intervals (CI). The adjusted model controlled for age, sex, race, BMI, SES, urine sodium excretion, total energy intake, alcohol intake, and fruit and vegetable consumption

In fully adjusted linear regression analysis (Table 3), dietary potassium showed no significant association with systolic blood pressure (B = − 0.494, 95% CI: −2.133 to 1.144, *p* = 0.554) but was inversely associated with diastolic blood pressure (B = − 1.339, 95% CI: −2.531 to − 0.147, *p* = 0.028). Twenty-four-hour urinary potassium demonstrated no significant associations with either systolic blood pressure (B = 0.476, 95% CI: −0.747 to 1.699, *p* = 0.445) or diastolic blood pressure (B = − 0.164, 95% CI: −1.058 to 0.729, *p* = 0.718). Similarly, the sodium-to-potassium ratio was not significantly associated with systolic blood pressure (B = 1.187, 95% CI: −0.305 to 2.679, *p* = 0.119) or diastolic blood pressure (B = 0.789, 95% CI: −0.301 to 1.879, *p* = 0.156). Overall, only dietary potassium was significantly associated with blood pressure after adjustment, while urinary potassium and the sodium-to-potassium ratio showed no significant associations.

## Discussion

This study provides novel methodological and contextual evidence on potassium intake assessment among young South African adults by directly comparing dietary recall-based estimates with 24-hour urinary potassium excretion. Using a dual-method approach within a well-characterised African cohort, we demonstrate consistently low potassium intake across assessment methods and identify systematic underestimation at higher intake levels when using dietary data alone. These findings extend beyond descriptive intake data by highlighting strengths and limitations of commonly used potassium assessment methods, with important implications for nutritional surveillance and cardiovascular prevention strategies in low- and middle-income settings.

Based on both methods, over 90% of our study participants did not meet the WHO-recommended potassium intake (3510 mg/day), which protects against salt-induced hypertension. Although the median urinary potassium was slightly lower than dietary potassium, a marginally higher proportion of participants (2.5%) met the recommendation based on urinary potassium, likely reflecting differences in the distribution and measurement characteristics of the dietary assessment method [29].

These results align with findings from previous South African studies which reported that over 90% South Africans did not meet this daily intake recommendation [13, 14, 30]. Concerningly, poor potassium intake among our study participants exists against the backdrop of excessive salt intake (9.3 g/day), highlighting the persistently high prevalence of hypertension in South Africa [3].

By racial comparison, White participants in our study had relatively higher daily potassium intake, including lower salt intake, than their Black counterparts, as well as higher fruit and vegetable consumption, which is one of the primary sources of potassium [10]. Other South African studies reported similar findings among White participants when compared to their counterparts [13, 30]. These findings are grounded in poor dietary practices and food insecurities among Black South African communities [31, 32], which often manifests through earlier hypertension onset [33]. Food insecurity has been directly associated with low potassium intake [34]. Participants from the low SES class also had the lowest potassium intake according to the two methods, reflecting the above vulnerabilities in this group [35], including being prone to the onset of hypertension development [36].

Agreement between dietary and urinary potassium intake was evaluated using Bland-Altman analysis. The plot demonstrated reasonable consistency between the two methods at lower intake levels, where most participants clustered, but agreement weakened at higher reported intakes, with increasing dispersion and several observations falling outside the limits of agreement, indicating proportional bias. The limits of agreement between dietary potassium and urine potassium were wide (− 2745.90 to 2868.48 mg/day), with an agreement span of approximately 5,600 mg/day, indicating substantial variability between the two methods at the individual level. This wide variability may partly reflect biological day-to-day variation in potassium excretion, as 24-hour urinary excretion exhibits substantial intra-individual variability; accordingly, reliance on a single 24-hour urine collection is well recognized as insufficient to accurately reflect habitual intake [21, 37]. Furthermore, dietary assessment methods may be affected by reporting errors and incomplete capture of dietary potassium intake [29], which could also contribute to disagreement at the individual level between the two methods.

Despite the individual-level variability, the mean difference between methods was small and statistically insignificant (61 mg/day; *p* = 0.19), with dietary potassium intake marginally exceeding urinary excretion. The noted direction is biologically plausible given incomplete renal excretion of potassium [28, 38, 39]. Both methods consistently indicated that many participants fell well below the recommended potassium intake threshold, leading to agreeable conclusions regarding widespread potassium inadequacy among South African young adults. Similar patterns of limited individual-level agreement between dietary and urinary potassium assessments have been reported previously, supporting the interpretation that dietary methods may be more suitable for population surveillance than for precise individual intake estimation [40–42].

After adjustment for potential confounders, only dietary potassium intake was inversely associated with diastolic blood pressure, supporting a well-established relationship between potassium and blood pressure [9, 43]. The effect size was modest (approximately 1–2 mmHg); however, with meaningful implications at the population level and long-term cardiovascular risk [44]. The observed limited association may reflect the young, predominantly normotensive nature of our study population, where restricted variability in blood pressure values may attenuate detectable associations. Previous studies have similarly reported weaker and less consistent potassium-blood pressure relationships in younger populations [45, 46]. Therefore, while the present findings suggest that potassium intake may play a modest role in the regulation of blood pressure during early adulthood, they may also highlight that the influence of potassium on blood pressure may vary according to population characteristics. Nevertheless, promoting adequate potassium intake remains important given its established role in cardiovascular health and its interaction with dietary sodium exposure [9].

### Strengths and limitations

To our knowledge, this is the first study in South Africa to assess potassium intake using both dietary assessments and 24-hour urinary analysis among young Black and White adults and across different SES backgrounds. This dual-method approach strengthened our ability to quantify potassium intake and to identify subgroups potentially at greater risk of salt-induced hypertension. Dietary intake was assessed using three 24-hour diet recalls supplemented with urinary potassium measurements, improving the accuracy of intake estimation. However, several limitations should be acknowledged.

After excluding participants with incomplete dietary and urinary potassium data, the final analytic sample contained proportionally more White than Black participants than the original cohort. Comparison of included and excluded participants revealed significant differences in sex, race, and SES, with excluded participants more likely to be female, Black, and of lower SES (Supplementary Table S1). Although sex, race, and SES were included as covariates in the multivariable regression models to account for potential confounding, this adjustment does not eliminate the possibility of selection bias due to differential exclusion.

Additionally, dietary intake relied on self-report, which is subject to recall bias, although this limitation was mitigated by excluding implausible energy reporters (> 30 000 kJ/day or < 3 000 kJ/day). Although a 24-hour urinary excretion is commonly used as a reference method for estimating potassium intake [20], it is subject to substantial within-person day-to-day variability [21]. Consequently, reliance on a single 24-hour urine collection may not accurately capture habitual potassium intake at the individual level [37] and represents a limitation of the present study.

Finally, SES was assessed using a modified Kuppuswamy socioeconomic scale, originally developed and validated in India [25]. While the scale incorporates widely used indicators of socioeconomic status and has been previously applied in the African-PREDICT study [27], its applicability to the South African context has not been formally validated. This may limit the comparability of SES classifications with those derived from locally developed socioeconomic measures.

## Conclusion

Both methods used in our study indicate that potassium intake was markedly low among young adults in South Africa, with particularly low intakes observed among Black participants and individuals from low-SES backgrounds. Comparison of dietary and urinary assessment methods showed limited individual-level agreement but consistent population-level conclusions about widespread potassium inadequacy. In unadjusted models, the sodium-to-potassium ratio was positively associated with both systolic and diastolic blood pressure; however, these associations were lost after adjustment. In the adjusted models, dietary potassium remained inversely associated with diastolic blood pressure, while no associations were observed for urinary potassium or the sodium-to-potassium ratio.

These findings support the utility of dietary assessment for population-level potassium surveillance while highlighting the need for cautious interpretation at the individual level, particularly among individuals with higher potassium intakes, where agreement with urinary potassium was less consistent. Collectively, the results underscore the importance of equity-focused strategies to improve potassium intake in South Africa and the use of context-appropriate assessment methods to inform CVD prevention policies.

## Supplementary Information

Supplementary Material 1.

## Data Availability

Data are available from the corresponding author upon reasonable request. Individual level data are restricted due to ethical and privacy requirements.

## Abbreviations

African-PREDICT: African Prospective study on the Early Detection and Identification of Cardiovascular disease and Hypertension
BMI: Body mass index
CVD: Cardiovascular diseases
SES: Socioeconomic status
