# Supplementary material for: Potassium intake assessed by dietary recall and urinary excretion in young South African adults: findings from the African-PREDICT study
Source: BMC Public Health. 2026 Jul 21;26:2973. doi: 10.1186/s12889-026-28605-5 (PMC13670738; doi:10.1186/s12889-026-28605-5)
Supplement: Supplementary file 1 [file 12889_2026_28605_MOESM1_ESM.docx]

**Supplementary Table 1**. Comparison of participants included in the final analytic sample and participants excluded due to incomplete dietary and urinary potassium data.

| **Characteristics** | **Included (945)** | **Excluded (254)** | **p-value** |
| --- | --- | --- | --- |
| **Age**, median (IQR) | 25 (5) | 24 (5) | 0.282 |
| **Sex** | | | |
| Female, n % | 472 (49.9%) | 149 (59.6%) | 0.007 |
| Male, n % | 473 (50.1%) | 101 (40.4%) |  |
| **Race**, n % | | | |
| Black | 429 (45.4%) | 175 (70.0%) | < 0.001 |
| White | 516 (54.6%) | 75 (30.0%) |  |
| **SES** | | | |
| Low SES | 352 (37.2%) | 123 (49.2%) | 0.002 |
| Middle SES | 279 (29.5%) | 65 (26.0%) |  |
| High SES | 314 (33.2%) | 62 (24.8%) |  |

**Table Caption:** Values are presented as median (IQR) or number (%). Age was compared using the Mann–Whitney U test, while sex, race, and SES were compared using chi-square tests. Sample sizes may vary slightly across variables because of missing data.
